# Recent Misadventures under Anæsthetics

**Published:** 1906-12-08

**Authors:** 


### SPECIAL ARTICLE.

### RECENT MISADVENTURES UNDER ANÆSTHETICS.

### DANGERS OF WRONGFULLY ATTRIBUTING SURGICAL FATALITIES TO ANÆSTHETICS.

Some surgeons are prepared to go very far in attributing deaths under operations to deaths from anæsthesia. An extraordinary instance of this is recorded of Dr. Faure, who, having removed the first two ganglia of the cord of the sympathetic on one side of the neck for the cure of ex-ophthalmic goitre, was setting to work on the third ganglion when the patient died. Instead of attributing the death to direct operative interference with the enervation of the heart, the operator declared that the removal of the sympathetic ganglia was in no way accountable for the death of the patient, but that the patient really died from the anæsthetic. Such a statement may have misled some other surgeons to try the same operation, and fatalities of a similar kind under similar circumstances have occurred. One London surgeon removed (the patient being anæsthetised with gas and ether) the left cervical cord completely, and then partially removed the right. The patient recovered from the anæsthetic, but died soon afterwards, having the classical signs of the removal of these ganglia well marked.

### More Details Required.

The manner in which deaths under anæsthetics are notified tends to throw undeserved odium on anæsthetists. No details as regards the posture of the patient, the precautions for the avoidance of blood entering the trachea, or any indication of the respiration being embarrassed or any post-mortem examination of the lungs recorded. A medical periodical reports as follows: "At an inquest in Nottingham on November 22, after the death of a man, aged 20, who died at the General Hospital, it was testified that chloroform was administered for the removal of a small growth from the nose. The operation lasted thirteen minutes, and it was not until it had been completed and chloroform had ceased to be given for two minutes that the patient's heart suddenly failed." In another case it is stated that "an inquiry was held in Liverpool on November 2 into the circumstances attending the death at the Royal Infirmary of a female patient, aged 50. An abdominal operation had been performed, ether being the anæsthetic administered. After the completion of the operation, and when the anæsthetic was being no longer administered, the patient had an attack of violent vomiting, and shortly afterwards died."

It seems that in both these cases it is complacently assumed that the fatal occurrence was in some way or other connected with anæsthesia. In former days skilled anæsthetists were few and far between. Now they are as thick as leaves in Vallombrosa, and it is rather a reflection on the institutions in which they have been trained to constantly ascribe blame to the anæsthesia for every misadventure during or subsequent to any operation. The evidence usually placed before juries in these cases should be fuller and should contain definite information derived from post-mortem examination. The surgeon should take his share of the blame, or else state conclusively in what way the anæsthetic contributed to the misadventure.

### A Misadventure at St. Thomas's Hospital Under Chloroform.

This is well exemplified by the following notes of a case upon which an inquest was held in St. Thomas's Hospital on October 22. The patient was a thin, nervous man, aged 36, a stationer of good general condition, except for an asthma of many years' standing. He had had nasal polypi removed under chloroform at St. Thomas's Hospital many years ago, and did well. On Wednesday, October 17, 1906, he was admitted into St. Thomas's Hospital for a further removal of nasal polypi; both nostrils were full of polypi, which were hanging into the throat.

The operation was at 2.40 p.m. The assistant-house physician gave evidence to the effect that he began to give chloroform by pouring it on to lint, a few drops at a time, from a drop-bottle. The patient was about five minutes "going under." About one dram was used in this way, and then the narcosis was continued with a Junker's vapour inhaler. When the operation had been proceeding for about ten minutes the patient went blue, and the breathing became embarrassed. Three drams of chloroform in all had then been used. The anæsthetic was discontinued and the breathing improved. There was not much bleeding. Some growths had been removed from the left nostril. The operation was not quite completed when the anæsthetist was instructed by the operator to discontinue the anæsthetic. The patient was quite unconscious and fully under the influence of the anæsthetic. He again became blue in the face, and the respiration ceased. The operator ceased operating, artificial respiration was resorted to, strychnine and ether injected by a nurse (!), the battery applied to the chest. He became bluer, breathed three times, and died at about 3 p.m.—*i.e.* about twenty minutes from the time of beginning to administer the anæsthetic. Artificial respiration was kept up for half an hour.

The position of the patient upon the operating-table was on the back, with the head turned to the right for the convenience of the operating surgeon, so that blood does not "*fall back into the larynx*," but *it may be drawn in by inspiration*. Whatever position the patient was in the same thing might have happened (!) Such was the evidence of the anæsthetist.

The following are notes of the post-mortem examination made by Ludwig Freyberger, M.D.:—Externally: The body was that of a thin and slender man. Internally: There were two or three polypi left hanging into the throat. Both bronchial tubes were blocked by blood. Left lung: The blood extended into the finer tubes, leaving hardly any lung tissue available, rendering it totally useless. Right lung was similarly blocked with blood, leaving about half the lung for respiratory purposes. Heart was small and friable. Mitral valve was thick and narrowed. Aorta was patchy. Liver and spleen congested. Kidneys: Both chronic Bright's disease. Stomach and bowels healthy. "In my opinion," concludes Dr. Freyberger, "death was due to suffocation consequent upon blood entering the windpipe and becoming fixed in the bronchial tubes." Verdict: Accidental death.

### A Misadventure at the Victoria Hospital, Chelsea, under Ethyl Chloride.

We are indebted to Dr. J. B. Copland for the following very complete account of a death which occurred at the Victoria Hospital, Chelsea, during the administration of ethyl chloride:—

G. E. G., age five, a hospital out-patient, first came under my treatment about a month ago. His symptoms were cough and considerable adenitis on either side of the neck. He was rather a "torpid" boy, both in speech and action. An examination showed no physical signs in the chest to account for the cough, and the cardiac action was regular and the sounds normal. I noticed that his tonsils were moderately enlarged, and that the nasopharynx was almost filled with a mass of adenoid growths. After three weeks' treatment he improved greatly constitutionally, and the glandular enlargements had much diminished in size. However, the cough continued, accompanied by epistaxis and some deafness, mouth-breathing, etc. It was decided to remove the tonsils and adenoids, a laxative was ordered, his parents were given suitable directions, and a morning was appointed for the operation. He was apparently quite well at the time, and the mother, being asked if the instructions of the paper had been followed, replied "Yes." No cardiac disease was detected previously to the anæsthetic. Knowing me well, he was not at all disturbed by the preparations, and there was very little increase in the rapidity of the heart's action. The patient was flat on the table, bare to the waist except for a mackintosh apron tied loosely round the neck. Ethyl chloride was given by the usual closed method: mask with pneumatic pad and bag with junction-tube having a hole for the spraying in of the anæsthetic. There was no residual ethyl chloride in the bag, as he was the first case to be operated on. I sprayed in three c.c. of Duncan Flockhart and Co.'s ethyl chloride, but some of this escaped, as the junction of the bag and tube was loose. A gag was inserted and the face-piece applied. After a few breaths I removed it, allowing one breath of air. On reapplying for a few more breaths, the eyes, which had been oscillating, became fixed, and I withdrew the anæsthetic. The operator removed the left tonsil. The other, only slightly enlarged, was left, and the patient was turned on his side for curetting.

I then noticed that there was no attempt at respiration, and the face was becoming blue. The adenoids were removed in a few seconds, and after cold spongings of the face he was turned on his back, and artificial respiration started. This was maintained uninterruptedly for three hours and a half, until all signs of life had ceased.

During this time every form of stimulant was administered. Ether, strychnine, brandy, adrenalin, sal volatile, oxygen, hot flannels and bottles; table raised, rhythmic traction of tongue, massage of cardiac area, wool bound round the limbs; saline, coffee, and brandy per rectum, without any response, except an occasional flickering action of the heart. After two hours ten drops of brandy were injected into the heart muscle, and the face, which before had been patchy purple and white, became a uniformally good colour, and a distinct increase of muscular tone was noticed, especially in the jaw; the tongue which had been flabby, thin, and colourless, swelled, and there was even a little arterial hæmorrhage from the puncture wound of a piece of fishing gut inserted to assist in traction. This injection was repeated, but without the same effect.

Post-mortem examination shows a general increase of lymphoid tissue throughout the body. Thymus gland enlarged, lobe weight 1¼ oz., rises above sternal notch; percussion failed to suggest any enlargement. Thyroid, normal; spleen, weight 3 oz., showed very prominent malpighian corpuscles; mesenteric, peritracheal, and peribronchial glands all moderately enlarged. Mesentery also shows numerous pink hæmolymph glands up to two-thirds of an inch in diameter. Lungs: Bases, congested, no signs of phthisis. The blood-vessels contain much fluid blood. Bronchi contain blood and mucous on both sides as far as they can be opened by scissors. There are small dark patches scattered about the lower parts of the lungs. Tissue in general œdematous. A few old adhesions between lobes of the left lung. Heart shows a small puncture wound on the anterior surface of the right ventricle, otherwise normal.

Remarks by the anæsthetist: It will be noticed that the adenoids were removed after the child turned a bad colour. Operators have observed that the free flow of blood incidental to this action generally excites a return of the respiratory movements. The condition of the lungs is one often met with in the lymphatic or thymic state, *vide* Hare's text-book on medicine, pp. 750-751. The child was, in fact, *in status lymphaticus*, as stated by Dr. Jex Blake, pathologist to the hospital, at the inquest, although this was not recorded in the newspaper reports. The anæsthetic was used in the out-patient department of this hospital 1,385 times between October 12 of last year and the same date in this without any serious accident. At times the patients have "gone blue" and taken a few minutes to recover, but usually the recumbent position with cold sponging of the face is sufficient.

### The Posture of the Patient.

Such cases call urgent attention to dangers which have hitherto been attributed to chloroform, but which are in large part due to the carelessness of the operator in permitting blood during operations on the air passages to trickle down into the larynx and thence into the bronchi and lungs. The upright posture seems better adapted for ethyl chloride, because the anæsthetist can readily push the head forward and permit the blood to issue from the mouth and nostrils. Some operators, at the Central London Throat Hospital, form with the finger a kind of lingual gutter permitting the blood to flow freely forward out of the mouth. It is almost unnecessary to add that with an operator adopting this method of removing tonsils and adenoids it is a rare thing to find sickness or vomiting after the administration of ethyl chloride.

### Alarming Case under Nitrous Oxide.

Another alarming case, though fortunately not fatal, occurred recently at the London Hospital during an operation for the removal of adenoids. The anæsthetic was nitrous oxide gas. During the administration the patient was seen to grow livid, and respiration ceased. It was conjectured that some of the adenoid tissue had caused a spasm of the glottis. Both administrator and operator were skilled and experienced, and the operation was done with the patient in the upright position and in a natural light.

### Chloroform in Dentistry.

To a female patient, aged 31, who was advised to have 14 or 15 teeth extracted, her usual medical attendant administered chloroform upon a mask. As the patient was passing from the second to the third degree of narcosis, respiration and circulation stopped. The necropsy showed the heart to be fatty. It has been pointed out by every expert and physiologist that the dental chair is no place for chloroform inhalation. Chloroform is not quite an up-to-date method of anæsthesia for the removal of teeth, and its administration for this purpose by the family physician is out of place and fraught with danger to himself and his patient. It betrays a lack of familiarity with up-to-date anæsthetic methods.

### Misadventures through Improper Dietary.

In conclusion it is no uncommon thing for blame to be attached to the anæsthetist who administers chloroform to patients whose guardians have been strictly enjoined to avoid administering solid foods previously to an operation. The parents nevertheless give such patients quantities of milk, or, if they are older children, hard-boiled eggs, oranges, and chocolates may be given on the way to the hospital to fortify them for the operation. Many cases of this kind occur, of which the following may be taken to be a typical example. An infant twelve months old had been operated upon on three successive occasions, each time by the same operator and anæsthetised by the same anæsthetist under exactly similar conditions without any untoward consequence. But on the last occasion and after the chloroform had been removed, it was found that on laying the child on the left side the heart suddenly stopped, and vomiting took place. Artificial respiration was resorted to, and continued for ten minutes without effect. On the anæsthetist examining the air passages with his finger he removed a large piece of curdled milk from the epiglottis, and breathing recommenced. It appeared that the stomach of the child was full of curdled milk, and when turned on its left side the heart stopped as if it had received a severe blow. Artificial respiration naturally produced no beneficial effect until the air passages were cleared. The child nearly died, and if it had done so blame would have attached to the anæsthetist, although, really, the person who had administered the food, wrongfully, was the immediate cause of the misadventure.

